# Microarray-based genomic profiling and *in situ* hybridization on fibrotic bone marrow biopsies for the identification of numerical chromosomal abnormalities in myelodysplastic syndrome

**DOI:** 10.1186/s13039-015-0136-5

**Published:** 2015-05-28

**Authors:** Marian JPL Stevens-Kroef, Konnie M Hebeda, Eugène T Verwiel, Eveline J Kamping, Patricia H van Cleef, Roland P Kuiper, Patricia JTA Groenen

**Affiliations:** Department of Human Genetics, Radboud university medical center, P.O. Box 9101, 6500 HB Nijmegen, The Netherlands; Department of Pathology, Radboud university medical center, P.O. Box 9101, 6500 HB Nijmegen, The Netherlands

**Keywords:** Microarray, *in situ* hybridization, Karyotyping, MDS, Fibrotic, Bone marrow biopsy

## Abstract

**Background:**

Myelodysplastic syndromes (MDS) are a heterogeneous group of clonal hematological malignancies. In MDS patients with a fibrotic bone marrow the aspiration of cells often fails (dry-tap), which hampers standard karyotyping. Obtaining genetic data from these fibrotic marrows is therefore challenging, and up till now *in situ* hybridization applied to bone marrow biopsies is the only option. The microarray-based genomic profiling technology has already proven its value for bone marrow aspirates and peripheral blood samples, but has never been applied to the technically challenging bone marrow biopsies. We describe an approach for microarray-based genomic profiling on bone marrow biopsies and demonstrate its ability to obtain clinically relevant cytogenetic aberrations. In addition the data were compared with those obtained by *in situ* hybridization and karyotyping.

**Results:**

We have evaluated the success rate of microarray-based genomic profiling by studying twenty-one bone marrow biopsies (7 fibrotic MDS, 12 non-fibrotic MDS and 2 reactive), by microarray-based genomic profiling and *in situ* hybridization (12 of 21 cases). The data obtained with these techniques were compared with conventional karyotyping data on corresponding bone marrow aspirates. Of the 15 copy number aberrations that were detected by *in situ* hybridization, 13 were concordant with microarray-based genomic profiling and karyotyping, whereas two hybridizations were misinterpreted. In 20 of 21 patients, the data obtained by microarray-based genomic profiling and karyotyping were identical or differences could be explained by the presence of marker chromosomes, complex karyotypes, clonal heterogeneity or disease progression.

**Conclusions:**

We demonstrate that genome wide microarray-based genomic profiling performed on bone marrow biopsies has a similar success rate compared to *in situ* hybridization, and prevents misinterpretation of chromosomal losses as observed by FISH. In addition, equal to even higher resolutions were obtained with genomic profiling compared to conventional karyotyping. Our findings indicate that microarray-based profiling, even on bone marrow biopsies, is a valid approach for the identification of genetic abnormalities. This is a valuable substitution in cases of fibrotic MDS lacking cytogenetic results.

## Background

Myelodysplastic syndrome (MDS) is a heterogeneous group of hematopoietic neoplasms characterized by bone marrow dysplasia and ineffective hematopoiesis which causes peripheral cytopenias and a risk of progression to acute myeloid leukemia (AML). The diagnosis and classification of MDS is based largely on clinicopathological, morphological and cytogenetic findings. In addition, chromosomal abnormalities are found in 40-60% of the patients and prove helpful for determination of prognostic status with regard to survival and AML evolution [1,2]. For instance in the International Prognostic Scoring System (IPSS) for MDS, cytogenetic subgroups of outcome were defined as follows: “good” outcomes were associated with a normal karyotype, −Y alone, del (5q) alone, del (20q) alone; “poor” outcomes showed complex karyotypes (ie, ≥3 abnormalities) or chromosome 7 anomalies; and “intermediate” outcomes were associated with all other abnormalities [1]. Conventional karyotyping is the current standard screening for chromosomal abnormalities on metaphases. This approach can be problematic in cases with fibrotic bone marrow biopsies, often resulting in none or a non-representative bone marrow aspirate sample (dry-tap) which does not contain neoplastic blasts. Such a dry-tap is observed in 4–7% of the bone marrow examinations [3,4]. *In situ* hybridization (ISH) using targeted probes to detect recurrent chromosomal trisomies and translocations has been applied to bone marrow biopsies in only a limited number of studies in MDS [5-8]. The reliable detection of losses of chromosomes, and especially loss of chromosome segments is difficult in tissue sections, since nuclei are generally only partly present on the slide due to cutting of the tissue, which may result in false positive identification of chromosomal losses [5]. In addition, there can be a significant intermingling of neoplastic and non-neoplastic cells in MDS biopsies, resulting in difficulties in the identification of genetically abnormal neoplastic cells. The combination of fluorescence *in situ* hybridization (FISH) and the detection of immunophenotypic markers in formalin fixed paraffin-embedded (FFPE) sections of bone marrow can overcome the problem of a low percentage of malignant cells [7].

Genome wide profiling using bacterial artificial chromosome (BAC) libraries, oligonucleotide and single nucleotide polymorphism-based microarray platforms has been applied in a research or exploratory setting to profile the genomic alterations in patients with MDS and AML [9-13]. However, these microarray-based genome profiling approaches have only been applied to high-molecular weight DNA isolated from bone marrow aspirates or peripheral blood samples. The experience with DNA obtained from formalin-fixed paraffin embedded (FFPE) bone marrow biopsies is very limited, but this source would be helpful in MDS patients with bone marrow fibrosis resulting in a non-representative bone marrow aspirate and subsequent lack of cytogenetic data. The major disadvantage of DNA from tissue blocks is the fragmentation and degradation during the formaldehyde fixation and decalcification process.

To evaluate the diagnostic value of microarray-based genomic profiling, especially on fibrotic bone marrow samples, we have compared microarray-based genomic profiling and FISH or Chromogenic *in situ* hybridization on bone marrow biopsies with data obtained by karyotyping of corresponding bone marrow aspirate samples in MDS patients with and without extensive fibrosis in the bone marrow biopsy. Despite its limitation in detecting (recurrent) balanced translocations and the lower sensitivity, we demonstrate that, also in patients with a fibrotic bone marrow, microarray-based genomic profiling using DNA obtained from bone marrow FFPE-biopsies has a similar success rate as compared to *in situ* hybridization applied to bone marrow biopsies. In addition, microarray-based genomic profiling overcomes the false positive identification of chromosomal losses that occurred in FISH, can identify genomic abnormalities which are outside the scope of the ISH probes applied, and has a comparable or even higher resolution than karyotyping, since copy number abnormalities > 10 Mb in size can be easily detected.

## Results

### Interpretation criteria of microarray data and description of genomic profiles

Tissues from FFPE trephine biopsy specimens of 24 MDS patients and 2 reactive non-fibrotic cases, all with a successful cytogenetic result from a simultaneous bone marrow aspirate, were initially selected. The quality of the extracted genomic DNA was assessed by its ability to amplify of control gene fragments of 200 bp or more. However, three of the 26 FFPE specimens had to be excluded due to an insufficient quality and/or quantity of DNA. A microarray-based genomic profile was obtained from genomic DNA from the other 23 patients. Two additional samples had to be excluded because of the noise level of the obtained array profiles as measured by the quality score in Nexus. Therefore, successful genomic profiling could be performed in 21 of 23 profiled cases (91%). We have divided the patients in three groups (Table 1). Group 1 consisted of 7 non-fibrotic MDS cases with cytogenetic abnormalities. Group 2 were 7 MDS cases with availability of an abnormal karyotype despite bone marrow fibrosis. Group 3 included 4 non-fibrotic MDS cases, 1 AML case and 2 biopsies of reactive bone marrow, all with a normal karyotype except for a balanced translocation of chromosome 3 in one case. For comprehensive analysis and interpretation of the microarray-based genomic profiling the profiles of the 7 patients without copy number alterations (CNA) as determined by karyotyping (group 3), were evaluated to set up the interpretation criteria. In all cases that fulfilled the quality score in Nexus, small sized aberrations up to 10 Mb in size were observed, which we have considered as normal genomic variants or background noise. Therefore the threshold for identification of a CNA was set at 10 Mb. This threshold is in line with the resolution of karyotyping. For copy number gains of chromosome 19 the threshold was set at 30 Mb, since it has been reported that the high GC content of this chromosome can result in different amplification efficiency, and that hybridization of the amplified products can result in erroneously assigned alterations of chromosome 19 on BAC library-based array-platforms [14]. Only aberrations fulfilling the above criteria were included in the genomic profiles. Since MDS samples can harbor complex patterns of cytogenetic abnormalities (≥3 abnormalities) [1,2], the description of the obtained genomic profiles using the standardized ISCN 2013 [15] nomenclature system may result in complex reports. Therefore, we have chosen to convert the genomic array profiles into so-called microarray-deduced copy number karyotypes, adapted from the ISCN nomenclature for conventional karyotyping as has been proposed by Simons at al. [16], thus allowing a comprehensive comparison of microarray-based genomic profiling data to conventional karyotyping data.

**Table 1:** **In situ hybridization, microarray and cytogenetic data of the patients**

**Group**	**Patient ID**	**Sex**	**Age (years)**	**Diagnosis**	**Fibrosis**	**FISH (BM biopsy)**	**Array based karyotype (BM biopsy)**	**Karyotype (BM aspirate)**
1	4902	M	63	RCMD	no	not done	del(16)(q11q23)	46,XY,del(16)(q11q23)[4]/46,XY[6]
	4519	F	59	AML	no	loss 7 (44%)	del(3)(p24p22),del(3)(p14p12),del(4)(p15),dup(5)(p11),del(5)(q11q35),del(7)(p14p11), del (7)(q11q26),del(10) (q11q24),del(10)(q25q26),dup(22)(q11)	45,XX,del(5)(q13q33),-7[4]/46,XX[16]
	4898	F	42	RCMD	no	loss 7 (50%)	−7,+21,+22	45,XX,-7[4]/47,sl,+21,+22[5]/46,XX[1]
	4893	M	80	RAEB-1	no	trisomy 8 (50%)	+8	47,XY,+8[10]
	4895	F	59	RCMD-RS	no	trisomy 8 (23%)	+8	47,XX,+8[6]/46,XX[4]
	4896	M	72	RCMD	no	loss 8 (47%) loss 7 (50%)	del(5)(q21q31),-7	44,XY,del(5)(q15q33),-7,dic(15;17)(p11;p11),-18,der(21;22)(q10;q10),+2mar[cp8]/46,XY[2]
	4912	M	64	RAEB-2	no	not done	+8,+21	47,XY,+21[1]/48,sl,+8[9]
2	4522	M	57	RCMD	grade 3	loss 20q (32%) trisomy 8 (18%)	del(3)(p24q25),del(5)(q14q34),del(6)(p24q16), del(7)(q11q32),+8,del (20)(q11)	40~44,XY,-5,-6,-7,+8,-20,+4mar[cp7]/46,XY[3]
	4894	M	67	RCMD	grade 2	loss 20q (77%)	del(20)(q11q13)	46,XY,del(20)(q13)[10]
	4899	M	58	RAEB	grade 3	not done	del(2)(p23),del(4)(p12),del(4)(q12q13.2), del(5)(q14q35),del(7) (q21q36),del(12)(p12p13),del(20)(q11q13)	44~47,XY,-2,-2,-4,del(4)(q31),-5,-7,add(9)(q34),add(12)(p13),−13,-15,-16,-17,add(17)(p13),del(20)(q11),+3~7mar[cp8]/46,XY[2]
	4903	M	58	AML	patchy fibrosis	faillure	no CNA	44~47,XY,-2,-2,-4,-4,+6,-7,-9,-10,-13,-13,-17,add(17)(p13),−19,del(20)(q11),+21,+21,+4~8mar[cp8]/46,XY[2]
	4523	F	58	RAEB-2	grade 3	loss 7 (46%) loss 5 (29%)	del(5)(q14q33),-7	45,XX,t(4;17;20)(q31;q12;q13),del(5)(q13q33),-7[10]
	4520	M	70	RAEB-2	grade 3	loss 7 (61%) loss 5 (40%)	-Y,del(5)(q11q34),-7,del(17)(p13),-18	42~44,X,-Y,-5,-7,+?12,-13,add(17)(p13),-20,-21,+3mar[cp13]
	4521	F	59	RCMD	grade 2	loss 5q (19%)	del(4)(q24q26),del(5)(q13q35),del(6)(q11q21), del(7)(q11),del (17)(p13.1p13.3)	45~46,XX,add(1)(q32),del(5)(q?22q33),del(6)(p22),add(7)(q11),-10,-13,del(17)(p11),+2~3mar[cp10]
3	4904	F	60	RAEB-2	no	not done	no CNA	46,XX[20]
	4906	F	64	vit. B12 deficiency	no	not done	no CNA	46,XX[23]
	4897	M	71	hypoplastic AML	no	not done	no CNA	46,XY[20]
	4518	F	45	pure red cell aplasia	no	loss 7q (34%)	no CNA	46,XX[20]
	4890	M	58	RCMD	no	not done	no CNA	46,XY[20]
	4892	F	63	RCMD-RS	no	not done	no CNA	46,XX,t(3;3)(q21;q26)[11]
	4900	F	63	RAEB-1	no	not done	no CNA	46,XX[20]

RCMD = refractory cytopenia with multi lineage dysplasia, RAEB = refractory anemia with excess of blasts, RCMD-RS = RCMD with ringsideroblasts, AML = acute myeloid leukemia, no CNA = no copy number abnormality, BM = bone marrow.

### Comparison of microarray-based genomic profiling and *in situ* hybridization on bone marrow biopsies

**Figure 1:**
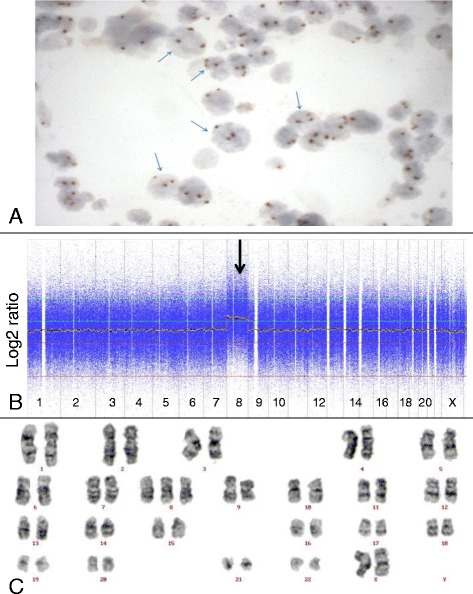
Trisomy 8 detected by Chromogenic *in situ* hybridization, karyotyping and microarray-based genomic profiling. Chromogenic *in situ* hybridization with probe for centromere of chromosome 8 (panel **A**), microarray-based genomic profile (panel **B**) and karyotype (panel **C**) of patient 4895, all indicating a trisomy 8.

Parallel microarray-based genomic profiling and *in situ* hybridizations using relevant probe-sets were applied to 12 of the 21 patients, which covers 15 different probe hybridizations. For an unbiased comparison the microarray and *in situ* hybridization data were analyzed in a fully blinded fashion. In four patients (4893, 4895, 4894 and 4523) concordant results were obtained by *in situ* hybridization and microarray-based genomic profiling (Figure 1). In six cases (4519, 4898, 4896, 4522, 4520 and 4521) additional genetic abnormalities were observed by microarray, while in two cases (4896 and 4518) *in situ* hybridization resulted in the identification of an additional chromosome loss, not observed by microarray and karyotyping. In one other case (4903) FISH was unsuccessful (Table 1). *In situ* hybridization and microarray-based genomic profiling were thus concordant in 13 of the 15 (87%) successful *in situ* hybridizations. These cases all involved the detection of a trisomy (cases 4893, 4895, 4522) or the losses of whole chromosomes 5 and 7 using FISH probe sets based on two probes in different colors (cases 4519, 4898, 4896, 4523, 4520).

For assessment of the detection limit of the microarray platform we have compared the genetic abnormalities as identified by microarray-based genomic profiling with those as determined by FISH on the same bone marrow biopsy. Abnormalities present in more than 18% of the cells as determined by FISH were readily detected by microarray, including 3 cases with a trisomy 8 (cases 4522 (18%), 4895 (23%) and 4893 (50%)), 3 patients with del(5q) (cases 4521 (19%), 4523 (29%) and 4520 (40%)) 2 patients with del(20q) (cases 4522 (32%) and 4894 (77%)) and 5 patients with loss of chromosome 7 (cases 4519 (44%), 4898 (50%), 4896 (50%), 4523 (46%) and 4520 (61%)).

### Comparison of microarray-based genomic profiling and karyotyping

**Figure 2:**
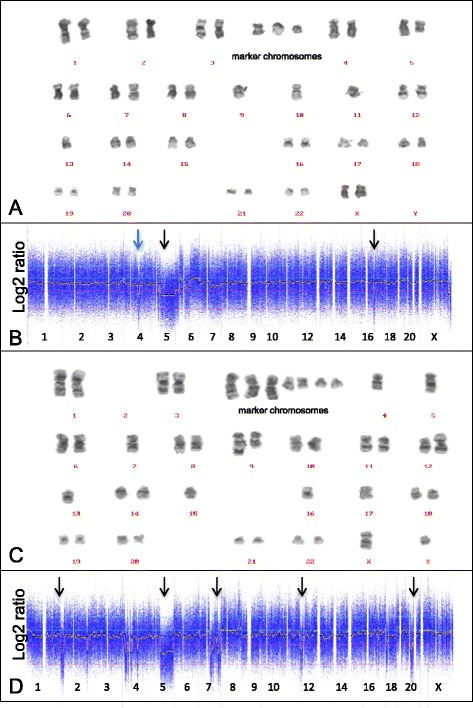
Comparison of karyotyping and microarray-based profiling in two patients with complex karyotypes. Karyotypes and microarray-based genomic profiles from patient 4521 (panel **A** and **B**) and 4899 (panel **C** and **D**). CNA observed with microarray-based genomic profiling are marked by arrows, including a 12 Mb focal loss of chromosome 4q24q26 (blue arrow).

In all 21 patients with successful karyotyping on the bone marrow aspirates microarray-based genomic profiling was successfully performed on the bone marrow biopsies (Table 1). No significant difference in genomic aberrations was observed between the MDS patients without (group 1) and with (group 2) fibrotic bone marrow. In 7 (pseudo) diploid patients (group 3) CNA were neither demonstrated by microarray-based genomic profiling nor by karyotyping. Conventional karyotyping on two patients revealed the presence of balanced genomic abnormalities, which as expected were not detected by microarray-based genomic profiling. One case (4892) without CNA involved the balanced t(3;3)(q21;q26), and in the other case (4523) the t(4;17;20) was present as additional abnormality. In seven patients the CNA obtained by microarray-based genomic profiling and karyotyping were exactly identical. In five other cases with complex karyotypes (4896, 4522, 4899, 4520 and 4521) there was no exact match between microarray-based genomic profiling and karyotyping (Figure 2). However, the discrepancy could be explained by the presence of marker chromosomes which are (in part) derived from missing chromosomes. In four of these patients (4522, 4899, 4520 and 4521) the loss of a chromosome or chromosome segment and the presence of marker chromosomes could be correlated with complex abnormal array profiles of the corresponding chromosomes or chromosome segments (Figure 2). In addition, all cases involved a so-called composite karyotype, indicating the presence of clonal heterogeneity. Due to clonal heterogeneity chromosome abnormalities may be present in a small fraction of the cells and remain undetected by microarray-based genomic profiling. One of the patients (case 4521) harbored a focal 12-Mb loss involving the 4q24 region which contains the in MDS patients recurrently mutated *TET2* gene [11], which was most likely missed by karyotyping due to its size below the cytogenetic resolution (Figure 2). In two patients there was a discrepancy between karyotyping and microarray-based genomic profiling. In one of these patients (case 4519) next to the del (5q) and loss of chromosome 7 as observed by karyotyping, additional genetic abnormalities were observed by microarray profiling. In this patient the microarray analysis was performed on a sample obtained 5 months later than the sample used for karyotyping, and might reflect the disease progression that was also demonstrated by histological and cytological analysis in this patient. The discrepancy in patient 4903 with a complex abnormal karyotype in the aspirate and a normal microarray-based genomic profile derived from the bone marrow biopsies may be explained by the sparse hematopoiesis present in the bone marrow biopsy of this patient, possibly resulting in a DNA-sample consisting of predominantly non-hematopoietic cells.

## Discussion

Currently karyotyping is the gold standard for the detection of prognostic relevant chromosomal aberrations in MDS [1,2]. However, its application is limited to dividing cells obtained from bone marrow aspirates. In MDS patients with fibrotic bone marrow, the marrow aspirate sample is often not representative (dry tap), and thus not suitable for karyotyping [3,4]. Although challenging, analysis of bone marrow biopsies by FISH or microarray-based genomic profiling could represent an alternative approach for detection of genetic abnormalities in these MDS cases with fibrotic bone marrow.

Numerous studies have demonstrated that FISH and microarray-based genomic profiling on cells obtained from bone marrow aspirates can identify genetic abnormalities [13,17-19]. However, despite the importance of the use of bone marrow biopsies for the diagnosis of hematological malignancies [20,21], the application of FISH on bone marrow biopsies has been barely investigated [5-8,22] and the application of microarray-based genomic profiling not at all. This may be related to technical limitations as a poor DNA quality due to DNA degradation during FFPE fixation, decalcification and tissue processing [22,23], resulting in relatively noisy array profiles [24,25]. Recently optimized sample preparation approaches involving the fixation and decalcification process, the use of Qiagen DNA extraction kit, and improved data analysis methods, have been recommended to improve the detection of CNA by genomic arrays on FFPE samples [24]. We have optimized the fixation and decalcification procedure. Despite these adaptations, we had to set the threshold at 10 Mb for the identification of CNA, which is much higher than required for high-molecular weight DNAs obtained from bone marrow aspirates or peripheral blood [26,27]. In addition the threshold for chromosome 19 abnormalities was increased to 30 Mb since GC-rich regions, which are abundantly present on chromosome 19, are less sensitive to DNA degradation (occurring in FFPE samples), leading to a more efficient PCR amplification and hence higher signal intensity. The recently introduced OncoScan array platform (Affymetrix) with the use of the molecular inversion probe strategy has shown to perform well with degraded DNA samples obtained from FFPE specimens [28-30].

We have evaluated the diagnostic power and limit of detection of microarray-based genomic profiling compared to FISH and Chromogenic *in situ* hybridizations on bone marrow biopsies. In 13 of the 15 successful *in situ* hybridizations concordant results were obtained by *in situ* hybridization and microarray-based genomic profiling (Table 1). In two other cases (4896 and 4518) *in situ* hybridization resulted in a false positive identified abnormality. In these two cases *in situ* hybridization was based on a single probe and suggested loss of chromosome segments (deletion 7q and loss of chromosome 8 respectively), which was not observed by microarray-based genomic profiling and karyotyping. As noted by others, the reliable detection of chromosome loss by FISH is difficult, since nuclei of cells in tissue sections are often sectioned [5]. In addition, the identification of balanced rearrangements and gain of chromosomes by FISH in tissue sections is more straight forward to detect because of a simpler probe configuration. This was confirmed in the present study were we have identified all three patients with a trisomy 8 by *in situ* hybridization correctly. Furthermore, the applied microarray platform exhibited a high limit of detection, i.e., CNAs present in at least 18% of the cells as determined by FISH could unambiguously be detected.

Microarray-based genomic profiling of non-fibrotic as well as fibrotic bone marrow biopsy samples demonstrated a high concordance with karyotyping on bone marrow aspirates, and discrepancies between both approaches could be explained by the complex karyotypes, the presence of marker chromosomes, the presence of balanced translocations, clonal heterogeneity or progression of the MDS. The only exception is a cytogenetically abnormal patient with a normal array profile (case 4903). The discrepancy may be explained by the sparse hematopoiesis in the biopsy, resulting in too few malignant cells present for microarray analysis, while cell culture for karyotyping, selective growth of aberrant blasts could have enabled detection of the cytogenetic aberrancies.

One important benefit of microarray-based genomic profiling as compared to FISH is its ability to detect additional chromosomal aberrations not detected by targeted probe-based assay. In four of the five patients with additional genetic abnormalities as compared to *in situ* hybridization, the microarray profile was indicative for a so-called complex karyotype which is associated with an adverse prognosis [2,31]. In addition a focal loss on chromosome 4q24q26 region containing the *TET2* gene as demonstrated by microarray-based genomic profiling in case 4521 could not be observed by karyotyping because it was below the level of cytogenetic resolution. The protein encoded by the *TET2* gene plays a key role epigenetic modification of the genome by DNA demethylation. The identification of this focal loss of the *TET2* gene is of clinical relevance since it has been demonstrated that this is a recurrent genetic abnormality in MDS patients [11].

## Conclusions

Our work represents the first genome wide microarray approach for the analysis for CNA in bone marrow biopsy samples in MDS patients. This is of clinical relevance for MDS patients with fibrotic bone marrows in whom cytogenetic data are lacking. In these patients microarray is able to identify cytogenetic abnormalities which are important parameters for the (revised) international prognostic scoring system for MDS patients. We demonstrate a high concordance of karyotyping on bone marrow aspirates and microarray-based genomic profiling on both non-fibrotic and fibrotic bone marrow biopsies in MDS patients. Although *in situ* hybridization can be applied to bone marrow biopsies, it is important to note that *in situ* hybridization for losses of chromosomes or chromosome segments may be misinterpreted due to technical limitations. Therefore this study demonstrates in MDS cases with failure to acquire a representative bone marrow aspirate that microarray-based genomic profiling eventually complemented with FISH for balanced prognostic relevant abnormalities (eg. 3q26/*MECOM* rearrangements) is a valid alternative approach to obtain information on clinical relevant cytogenetic abnormalities.

## Methods

### Patients

Twenty-six bone marrow biopsies from MDS patients with a successful cytogenetic result, were initially selected from the hospital archive. Of these 26 samples, 5 were excluded because of several reasons (as described under Results). For the remaining 19 MDS cases and 2 cases with a reactive non-fibrotic bone marrow, a good-quality microarray-based genomic profile was obtained, and these patients were further evaluated in this study. Seven MDS patients were selected because of an abnormal karyotype as determined by conventional cytogenetics on a simultaneous bone marrow aspirate (group 1). Seven other patients were selected because they had an abnormal karyotype and extensive fibrosis in the bone marrow biopsy (fibrosis grade 2/3 or 3/3) (group 2). Another 4 MDS, 1 AML and 2 reactive non-fibrotic BMB without copy number alterations (CNA) as determined by karyotyping were selected as controls (group 3) (Table 1). The diagnosis of MDS was made by combining histology, cytomorphology, clinical and cytogenetic data.

The bone marrow biopsies were fixed in Burckhardt pH 7.4 at room temperature, followed by decalcification in EDTA 10% pH 7.2 (room temperature) during 48 hrs. Post-decalcification tissue processing consisted of dehydration and paraffin embedding using either the Pathos Rapid Microwave Histoprocessor (Milestone, Sorisole, Italy) or the Shandon™ Excelsior™ ES Tissue Processor (Thermo Scientific, Waltham, MA, USA).

This study was approved by the institutional review board of the Radboud university medical center and in accord with the Helsinki Declaration 1975, as revised in 2008.

### Karyotyping

Hematopoietic cells from bone marrow aspirates were cultured and harvested for cytogenetic analysis by established methods. Chromosome aberrations were described according to guidelines of an International System for Human Cytogenetic Nomenclature [15].

### *In situ* hybridization

Fluorescence *in situ* hybridization (FISH) was performed based on the protocol described by Gerr et al. [32]. Briefly, 4 μm tissue sections were pretreated with 10 mM sodiumcitrate pH 6.0 using a microwave, digested with 200 U/ml pepsin/0.01 M HCl and fixated in 1% buffered formaldehyde. The commercially available probes LSI D7S485/CEP 7 (7q31/centromere 7), LSI EGR1/D5S721/D5S23 (5q31/5q15.2) and LSI D20S108 (20q12) (Abbott Molecular, Des Plaines, Illinois, USA) were used according to the manufacturer’s specifications. Signals were counted using an fluorescence microscope (DM4000B, Leica, Rijswijk, Netherlands). The homemade probe CEP 8 (D8Z2) labeled with biotin was detected using immunohistochemical staining (Chromogenic *in situ* hybridization). The immunohistochemical staining was performed by successive incubations with mouse-anti-biotine (Vector, Vector Laboratories, Burlingame, CA, USA), BrightVision Poly-HRP-Anti Ms/Rb/Rt IgG (Immunologic, Duiven, The Netherlands), and Bright-DAB (Immunologic, Duiven, The Netherlands) according to manufacturer’s specifications. Interpretation was performed as described [32] and least 100 cells were evaluated per case.

### DNA extraction and microarray-based genomic profiling

5–10 μm paraffin-embedded tissue sections were used to extract DNA using proteinase K treatment step according to the standard protocol. Subsequently, the DNA was treated with RNAase A (Qiagen, Venlo, The Netherlands) and was affinity-purified using QIAamp MinElute Columns (Qiagen, Venlo, The Netherlands). DNA sample concentration and quality were assessed by spectrophotometry and by PCR-amplification using the BIOMED-2 Control gene primer set [33]. Affinity-purified DNA samples (100 ng) that were able to amplify control gene fragments of 200 bp or more were used for whole genome amplification (ENZO diagnostics Inc., Farmingdale, NY, USA) for subsequent microarray profiling. Microarray-based genomic profiling was carried out using a Nimblegen human CNV 3 × 720 K whole-genome tilling array (Roche NimbleGen Inc., Madison, WI, USA) according to the manufacturer’s protocols. Each sample was referenced against a sex-matched non-tumor bone marrow biopsy sample. Data were analyzed using the NEXUS version 5 (Biodiscovery, Hawthorne, CA, USA) software package. The quality of the array hybridization was monitored by determination of the variance of difference between adjacent log ratio’s after excluding the outliers (Nexus QC score). Array hybridizations with a Nexus QC above 0.4 were excluded for further analysis. Abnormal segments were identified by the FASST2 segmentation algorithm with a significance threshold of 1.0 E-20, a maximum probe spacing of 1 Mb, and a minimum number of 10 probes for a segment. The log2 thresholds were set to 0.4, 0.12, −0.18, and −0.6 to distinguish between high copy gains, single copy gains, single copy losses, and homozygous losses respectively. Only segments fulfilling the above criteria and >10 Mb were identified as CNA.

### Ethical consent

This study is performed according to code for proper use of human tissue in the Netherlands as determined by the Federation of Medical Scientific Societies and in compliance with the Helsinki Declaration.

## Abbreviations

MDS: Myelodysplastic syndrome
AML: Acute myeloid leukemia
FISH: Fluorescence *in situ* hybridization
FFPE: Formalin fixed paraffin-embedded
CNA: Copy number alterations
